# Investigation of the Band Structure of Graphene-Based Plasmonic Photonic Crystals

**DOI:** 10.3390/nano6090166

**Published:** 2016-09-09

**Authors:** Pingping Qiu, Weibin Qiu, Zhili Lin, Houbo Chen, Yixin Tang, Jia-Xian Wang, Qiang Kan, Jiao-Qing Pan

**Affiliations:** 1 College of Information Science and Engineering, Huaqiao University, No. 668, Jimei Avenue, Jimei District, Xiamen 361021, China; 1511301022@hqu.edu.cn (P.Q.); zllin@hqu.edu.cn (Z.L.); 1400201017@hqu.edu.cn (H.C.); yixin_tang_2016@163.com (Y.T.); wangjx@hqu.edu.cn (J.-X.W.); 2 Institute of Semiconductors, Chinese Academy of Sciences, 35A, Qinghua East Road, Haidian District, Beijing 100086, China; kanqiang@semi.ac.cn (Q.K.); jqpan@semi.ac.cn (J.-Q.P.)

**Keywords:** graphene, photonic crystals, surface plasmon polaritons, nanocavity

## Abstract

In this paper, one-dimensional (1D) and two-dimensional (2D) graphene-based plasmonic photonic crystals (PhCs) are proposed. The band structures and density of states (DOS) have been numerically investigated. Photonic band gaps (PBGs) are found in both 1D and 2D PhCs. Meanwhile, graphene-based plasmonic PhC nanocavity with resonant frequency around 175 THz, is realized by introducing point defect, where the chemical potential is from 0.085 to 0.25 eV, in a 2D PhC. Also, the bending wvaguide and the beam splitter are realized by introducing the line defect into the 2D PhC.

## 1. Introduction

Photonic crystals (PhCs), first proposed by Yablonovitch [1] and John [2], have attracted great attention due to their unique properties, such as the self-collimation which can be applied to confine and guide light in photonic integrated circuits [3], the negative refraction which can be employed to focus light on a scale less than the square of light wavelength [4]. Furthermore, similar to the energy band gaps resulting from the electrons in a periodic potential, the photonic band gaps (PBGs) can also be obtained in medium with spatially periodical dielectric constant [5,6,7,8]. Light with a frequency within the PBGs is not allowed to propagate along the PhCs. However, conventional dielectric based PhC devices [9,10,11,12], usually in micrometer scale, are challenging to obtain a further size reduction due to the optical diffraction limit. Nevertheless, surface plasmon polaritons (SPPs), the electromagnetic waves propagating along an interface between a metal and a dielectric, enable the confinement of electromagnetic field to scales far below the optical diffraction limit [13]. This makes SPPs-based components significantly smaller than the wavelength [14]. However, noble plasmonic materials including gold and silver, are barely tunable and display enormous ohmic losses due to the evanescent characteristic of SPPs, which greatly limit their applications in nano-optical devices.

Graphene, a two-dimensional (2D) carbon atomic crystal arranged in a honeycomb lattice [15], has demonstrated exceptional properties in electronics and photonics [16,17,18], involving a number of applications such as field-effect transistors [19,20,21], photodetectors [22,23] and high-speed optical modulators [24,25], etc. Unlike the conventional plasmonics based on noble metals, graphene supported SPPs have demonstrated extremely high confinement, highly tunability via electrical gating and chemical doping [26,27,28], and relatively low loss resulting from long plasmon lifetime [29,30]. These extraordinary properties make graphene a promising candidate for plasmonic material with broad applications from terahertz to mid-infrared span [31,32,33,34].

One of the typical structures of graphene-based one-dimensional (1D) plasmonic PhCs is graphene micro-ribbon arrays. Early in 2011, Ju et al. reported the tunability of plasmon excitations and light-plasmon coupling at terahertz in graphene micro-ribbon arrays [35]. Then, Nikitin et al. investigated the transmission, absorption and reflection properties of graphene micro-ribbon arrays [36]. Bludov et al. demonstrated that polaritonic crystal can be realized with monolayer graphene periodically modulated by gate electrode [37]. Furthermore, Yan et al. studied the magnetoplasmons of graphene disk arrays of micrometer [38], where they found that the plasmon lifetime in graphene can be continuously tuned via magnetic field, and the tunability of mode splitting. In the same year, Thongrattanasiri et al. demonstrated the complete optical absorption property with similar structure [39]. Other properties of interest including transparency and band structure are investigated using graphene/insulator stacks [40,41]. However, all of the above mentioned research concerned the interaction between the graphene periodical nano structures and the normal incident light. In other words, the reported work did not analyze the density of states (DOS) properties of the plasmonic crystals, which are significant important in the performance of the future plasmonic emitters. Further, in plane propagation properties of plasmons along the 2D graphene plasmonic crystal have never been reported.

In this paper, we proposed graphene-based plasmonic PhCs with ultra-small size. Both the band structure and the DOS of 1D and 2D PhCs are numerically studied by using the finite element method (FEM) with Comsol Multiphysics Radio Frequency (RF) Module, version 4.3b, commercial software. Further, 2D PhC nanocavity was obtained by introducing point defect, where the optical field was well-confined in the defect via tuning the chemical potential of graphene in the defective region. Also, the bending wvaguide and the beam splitter are realized by introducing the line defect into 2D PhC. These results provide new insight into the designing of PhC devices, including PhC waveguides, PhC nanocavities, plasmonic filter, plasmonic switch, etc.

## 2. Simulation Methods and Models

**Figure 1:**
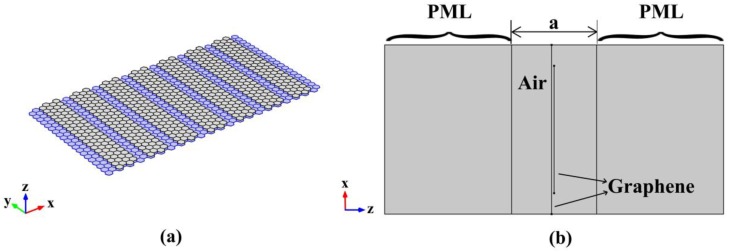
(**a**) The schematic view of the one-dimensional (1D) graphene-based plasmonic photonic crystal (PhC), where the blue denotes the bottom sheet graphene, and the grey denotes the graphene nanoribbons which modifies the plasmon propagating along the bottom sheet; (**b**) The computational window of 1D graphene-based plasmonic PhC with lattice constant a = 10 nm; The perfectly matched layer (PML) with width of 15 nm, is set up here as a non-reflecting boundary condition.

To investigate 1D graphene-based plasmonic PhC, double-layer graphene, constituted by two graphene sheets with an interval of 0.3 nm, is periodically arranged along x direction to form the 1D PhC in our model. Figure 1a displays a three-dimensional (3D) view of a schematic diagram of the 1D PhC. In Figure 1b, we use two line segments to represent the cross section of double-layer graphene with the same chemical potential of 0.9 eV. The bottom layer with width of 20 nm, which plays the role of guiding SPPs, is continuously arranged. Periodically discrete graphene nanoribbons with a width of 15 nm, which can be used to tune the plasmons in the bottom layer, are employed to form the top layer.

The graphene sheet is treated as an ultra-thin film that can be characterized by surface conductivity in our model. Therefore, the surface current density of graphene is defined as *J* = σ_g_*E* along the graphene sheet [42,43,44], where *E* is the electric field of the plasmon. The surface conductivity of graphene σ_g_, constituted by the interband electron transitions σ_inter_ and the intraband electron-photon scattering σ_intra_, is obtained from the Kubo formula [45,46,47,48],

(1) $$\sigma_{g}=\sigma_{\mathrm{int}ra}+\sigma_{\mathrm{int}er}$$

with

(2) $$\sigma_{\mathrm{int}ra}=\frac{-ie^{2}k_{B}T}{\pi h^{2}(\omega -i/\tau )}\left[\frac{\mu_{c}}{k_{B}T}+2\ln\left(1+\exp(-\frac{\mu_{c}}{k_{B}T})\right)\right]$$

(3) $$\sigma_{\mathrm{int}er}=\frac{-ie^{2}}{2h}\ln\left[\frac{2\;|\mu_{c}|\;-\;h\;(\omega -i/\tau )}{2\;|\mu_{c}|\;+\;h\;(\omega -i/\tau )}\right]$$

where μ_c_ is the chemical potential, *k_B_* is the Boltzmann constant, *T* is the temperature, ω is the angular frequency of the plasmon, and τ is the electron momentum relaxation time. In this paper, we set *T* = 300 K, τ = 0.5 ps.

**Figure 2:**
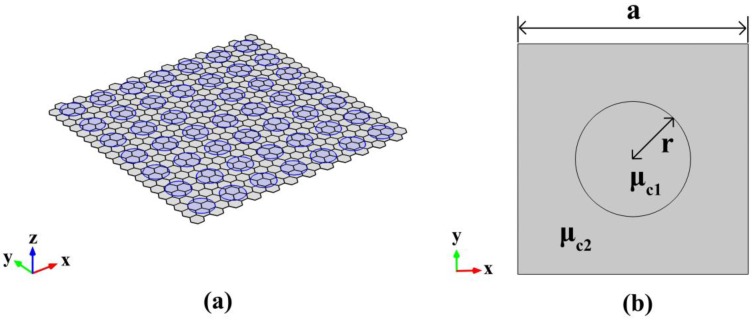
(**a**) Three-dimensional (3D) view of the proposed two-dimensional (2D) graphene-based plasmonic PhC. (**b**) The computational window of schematic diagram of graphene-based 2D plasmonic PhC, where lattice constant a = 20 nm, and r = 5 nm.

As for 2D plasmonic PhC, we use monolayer graphene with periodically modified chemical potential, to form the 2D plasmonic PhC with square lattice and lattice constant *a* = 20 nm. Figure 2a displays the 3D view of schematic diagram of 2D PhC, and as shown in Figure 2b, a circle with chemical potential μ_c1_, is surrounded by the same sheet of graphene with a different chemical potential μ_c2_. The graphene material can be characterized by effective index defined as *n_eff_* = 𝛽/*k_0_*, where *k_0_* is the wave number in free space. As for transverse magnetic (TM) mode, the propagation constants of SPPs supported by a single-layer graphene, can be expressed as [49,50,51,52]

(4) $$\beta =k_{0}\sqrt{1-{\left(\frac{2}{\eta_{0}\sigma_{g}}\right)}^{2}}$$

where η_0_ (377 Ω) is the intrinsic impedance of the free space. As far as band structure calculations are concerned, it is generally sufficient to consider the wave vector *k* around the edge of the irreducible Brillouin zone (IBZ: form Г to *X* to *M*) while ignoring the inner region, which can be justified from the symmetry of the structure.

Similar to the electronic density of states which experience significant modifications in a semiconductor, the periodic modulation of the dielectric constant results in a photonic DOS. Photonic DOS is defined as the number of the PhC Eigen-state inside the unit frequency range. The DOS is calculated by counting all allowed states within the frequency region of an infinitesimally small interval, which is expressed as follows [53,54]:

(5) $$N\left(\omega \right)=\sum_{n}\int_{BZ}d^{2}k\delta \left(\omega -\omega_{n}\left(k\right)\right)$$

here the multiplication by the delta-function $\delta \left(\omega -\omega_{n}\left(k\right)\right)$ corresponds to making the extract of the Eigen-states with the same frequency. After the extraction, the integration is carried out. This means summation of the Eigen-states with the same frequencies within one band. After integration, the summation over all the bands is obtained.

## 3. Results and Discussion

**Figure 3:**
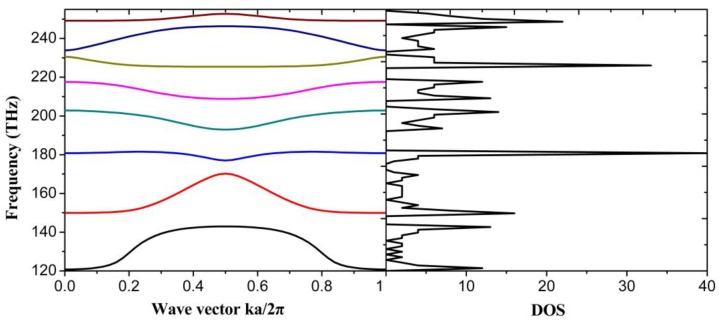
Band structure (**left**) and density of states (DOS) (**right**) of graphene-based plasmonic 1D PhC with chemical potential of 0.9 eV, and lattice constant a = 10 nm.

The periodicity of PhCs results in PBGs where no electromagnetic modes are allowed to have frequencies in the gaps. To obtain the band structure of plasmonic PhC, we need to iterate Eigen-frequency in each k point precisely due to the frequency dependent dielectric constant which results in the analytical solutions hardly being acquired. Figure 3 displays the band structure of 1D plasmonic PhC, and the corresponding DOS, with frequency from 120 THz to 255 THz. There are several PBGs from 142.9 THz to 149.9 THz, 170.2 THz to 177.1 THz, 181.5 THz to 192.9 THz and so on, where there are almost zero photonic DOS which correspond to the absence of the Eigen-states within corresponding frequency range.

**Figure 4:**
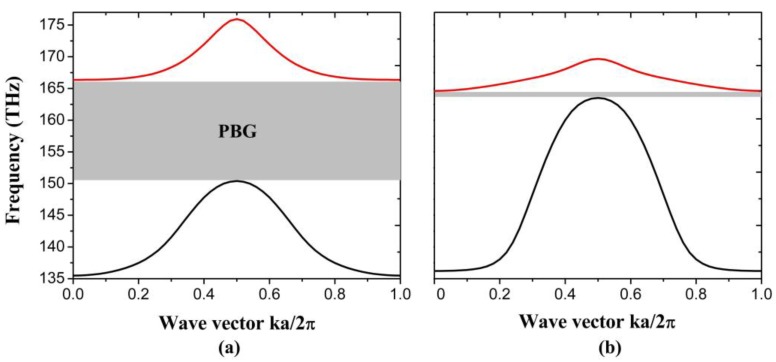
The first two bands of 1D PhC: (**a**) graphene nanoribbon with width of 13 nm; (**b**) graphene nanoribbon with width of 17 nm.

Furthermore, we modify the width of graphene nanoribbon in the top layer to see how the band structure changes. Figure 4a,b show the first two bands corresponding to the graphene nanoribbon with width of 13 nm and 17 nm respectively. The two bands cover the frequency range from 135.5 THz to 150.4 THz and 166.4 THz to 175.9 THz when we set the width of graphene nanoribbon as 13 nm, which displays a large PBG of 16 THz. However, when we increase the width of nanoribbon to 17 nm, the PBG can only cover frequency range from 163.5 THz to 164.6 THz. It can be seen that both the band position and bandwidth can be effectively tuned via modifying the width of graphene nanoribbon.

**Figure 5:**
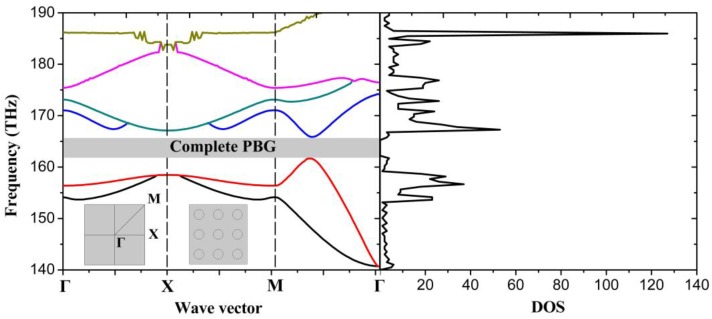
Band structure (**left**) and DOS (**right**) of graphene-based plasmonic 2D PhC with lattice constant a = 20 nm. The left inset shows the Brillouin zone, with the irreducible zone from 𝛤 to X to M. The right inset shows a view of the periodically modified chemical potential with μ_c1_ = 0.9 eV, μ_c2_ = 0.6 eV, and circular radius r = 5 nm.

In the same way as for 1D plasmonic PhCs, the band structure of 2D plasmonic PhCs can be calculated for a square lattice of 2D PhC with modified chemical potential. Figure 5 displays the band structure of graphene-based 2D plasmonic PhC with μ_c1_ = 0.9 eV, μ_c2_ = 0.6 eV, and the corresponding DOS. At some specific frequencies, the band gaps are overlapped forming so-called complete PBG with frequency range from 161.7 THz to 165.9 THz, which means that plasmons will not propagate inside the PhC at any angle. Moreover, the DOS within PBG is almost zero, which means there is no allowed Eigen-state in this frequency range.

**Figure 6:**
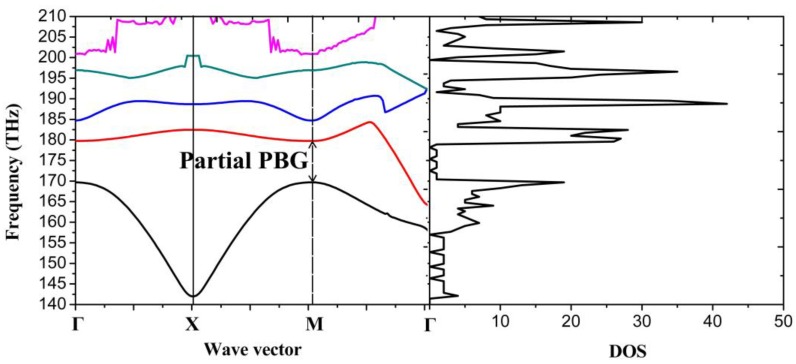
Band structure (**left**) and DOS (**right**) of graphene-based plasmonic 2D PhC with chemical potential μ_c1_ = 0.6 eV, μ_c2_ = 0.9 eV, circular radius r = 5 nm, and lattice constant a = 20 nm.

As it can be seen from Figure 6 with μ_c1_ = 0.6 eV, μ_c2_ = 0.9 eV, within all the investigated frequency ranges at least one Eigen-state exists, so no complete PBG appears. The first band covers the frequency range from 142.0 THz to 169.7 THz, the second band covers the frequency range from 164.1 THz to 184.3 THz, etc. However, considering the band structure, it is demonstrated that the PhC has wide partial PBGs at some certain propagation directions. For example, there are no Eigen-frequencies at the point M within the frequency range from 169.7 THz to 179.7 THz, which means that the light with frequency in this range propagating in the corresponding direction is forbidden.

**Figure 7:**
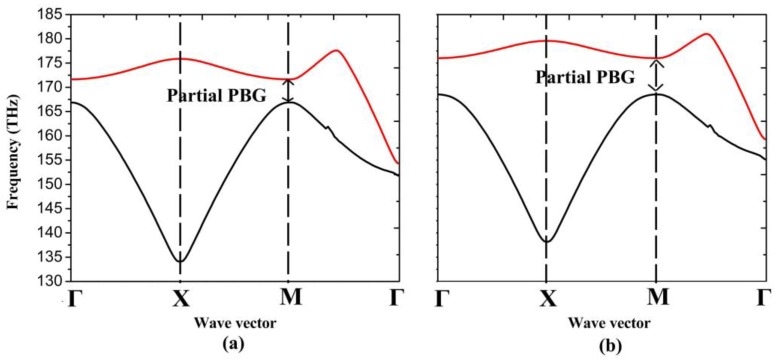
The first two bands of 2D PhC: (**a**) μ_c1_ = 0.6 eV, μ_c2_ = 0.8 eV; (**b**) μ_c1_ = 0.6 eV, μ_c2_ = 0.85 eV.

In order to tune the partial PBG (shown in Figure 6), we modify the chemical potential of the circle. As it can be seen from Figure 7a,b, the partial PBGs cover the frequency range from 166.9 THz to 171.6 THz and 168.5 THz to 176 THz, when we set μ_c2_ as 0.8 eV and 0.85 eV, respectively. Both the band position and bandwidth can be tuned distinguishedly.

## 4. Applications of Graphene Based Plasmonic Photonic Crystals (PhCs)

**Figure 8:**
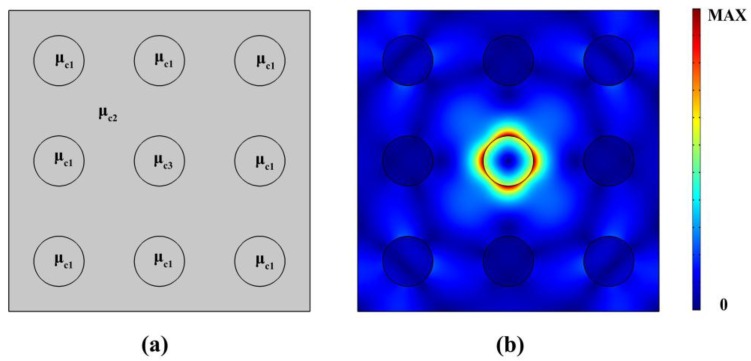
(**a**) Schematic of PhC nanocavity with ultra-small size of 60 × 60 nm, μ_c1_ = 0.6 eV, μ_c2_ = 0.9 eV; (**b**) The normalized electric field distribution of the resonant modes of the defect, with μ_c3_ from 0.085 eV to 0.25 eV.

As an application of graphene based plasmonic PhC proposed in this paper, the properties of the nanocavity formed by the point defect. To obtain PhC nanocavity, we introduce point defect into 3 × 3 PhCs, i.e., modifying the chemical potential of the central circle in Figure 8a. Figure 8a displays the schematic of the nanocavity with μ_c1_ = 0.6 eV, μ_c2_ = 0.9 eV, and the chemical potential of the defect is μ_c3_. As it can be seen from Figure 8b, the PhC nanocavity, with a light field well-confined within the defect region, can be realized.

**Figure 9:**
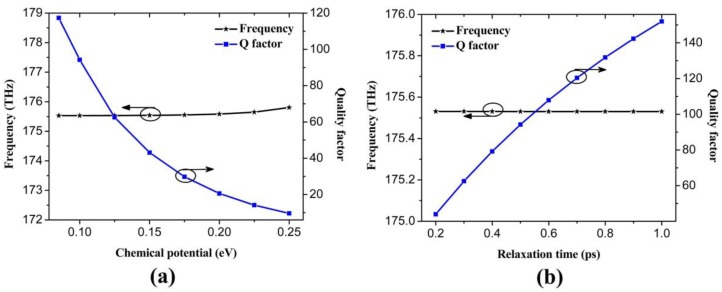
(**a**) Resonant frequency and Q factor as a function of chemical potential of the point defect in 2D PhC, where μ_c3_ is from 0.085 eV to 0.25 eV with μ_c1_ = 0.6 eV, μ_c2_ = 0.9 eV; (**b**) Resonant frequency and Q factor as a function of relaxation time with μ_c1_ = 0.6 eV, μ_c2_ = 0.9 eV, μ_c3_ = 0.1 eV.

Figure 9a plots the resonant frequency and quality (Q) factor for the resonant modes with the defect chemical potential μ_c3_ from 0.085 eV to 0.25 eV. It can be seen that the resonant frequencies are around 175 THz where the partial PBG exists (shown in Figure 5), which means that we have successfully introduced a point defect into the earlier proposed plasmonic 2D PhC, resulting in the PhC nanocavity. However, with the increasing of chemical potential of the point defect, the Q factor of the nanocavity decreases from 117 to 9.6. For a given frequency, the momemtum of the plasmon supported by graphene is dependent on the chemical potential. In the proposed nanocavity, when chemical potential increases, it becomes close to the chemical potential outside the cavities, so the momentum became more matched to the enviroment. The plasmon turns to escape from the cavities resulting from the increasing radiation loss. Eventually, the Q factor dicreases. Figure 9b displays the resonant frequency and Q factor versus the relaxation time. The resonant frequency remains around 175 THz, but Q factor increases from 44 to 152 with the increasing relaxation time from 0.2 to 1 ps. This can be attributed to the reduction of the Ohmic absorption of the plasmons when the relaxation time of electron momentum increases. Note that the typical mode area of the nanocavities is in the order of 10^−5^(λ_0_)^2^ (λ_0_ is the free space wavelength), which is comparable to the counterpart of the graphene coated InGaAs nanowire, and much smaller than the conventional cavities [31]. This nanocavity might be a fundermental component in the future high density integrated plasmonic circuit technique.

Practically, the means of fabrication of the proposed nano-cavity can follow the method proposed in Reference [34], where the graphene monolayer sits on a SiO_2_ on Si substrate. The thickness of the SiO_2_ layer is periodically modified to get the chemical potential of the graphene periodical variation when external electric field is applied. So the plasmonic crystal structure is achieved. When the thickness of one site of SiO_2_ is different from the regular value, the different chemical potential is obtained, and finally the nano-cavity is formed.

**Figure 10:**
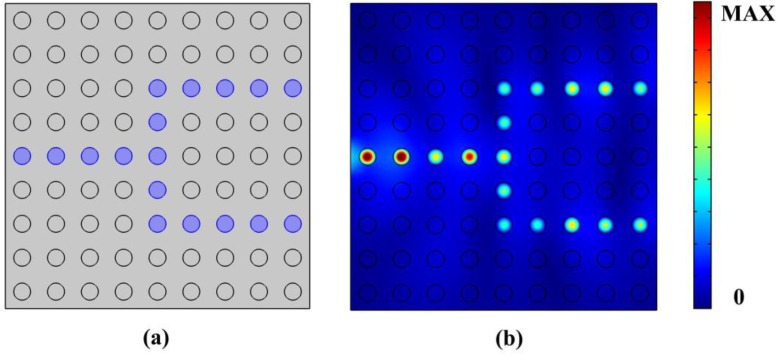
(**a**) Schematic of PhC beam splitter; (**b**) Normalized magnetic distribution of the PhC beam splitter with μ_c1_ = 0.6 eV, μ_c2_ = 0.9 eV, μ_c3_ = 0.3 eV, operation frequency of 9.8e13 Hz.

Another application of the 2D PhC is the beam splitter and bending waveguide. We introduce a line defect into 9 × 9 PhC. Figure 10a displays the schematic of the beam splitter with μ_c1_ = 0.6 eV, μ_c2_ = 0.9 eV. The path full of blue circles with chemical potential μ_c3_ of 0.3 eV represents the line defects which we used to realize the PhC beam splitter. Figure 10b shows the normalized magnetic distribution of the beam splitter with operation frequency of 9.8e13 Hz, where it can be seen that light was exactly transmitted through the routes we designed, and can also be well-confined within the defective region.

**Figure 11:**
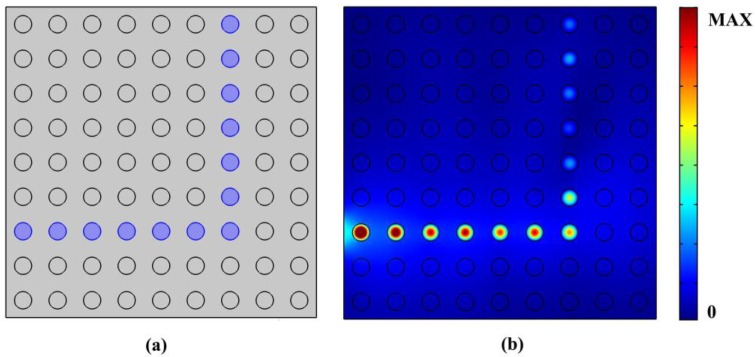
(**a**) Schematic of PhC bending waveguide; (**b**) Normalized magnetic distribution of the PhC bending waveguide with μ_c1_ = 0.6 eV, μ_c2_ = 0.9 eV, μ_c3_ = 0.3 eV, operation frequency of 9.8e13 Hz.

Figure 11a shows a bending waveguide with a line defect in the same crystal structure in Figure 10. The path full of blue circles with chemical potential μ_c3_ of 0.3 eV represents the line defects. Figure 11b displays the normalized magnetic distribution of the bending waveguide with a frequency of 9.8e13 Hz, where it can be seen that a 90 degree bending of waveguide with high confinement is realized by the proposed structure.

## 5. Conclusions

In summary, graphene-based 1D and 2D plasmonic PhCs are proposed, where the band structure and the DOS are numerically investigated. Several PBGs of 1D PhC are found, and there is no Eigen-state within the frequency range where PBGs exist. Meanwhile, we have found complete PBGs with frequency range from 161.7 THz to 165.9 THz for 2D PhC with μ_c1_ = 0.9 eV, μ_c2_ = 0.6 eV, and partial PBGs with frequency range from 169.7 THz to 179.7 THz for 2D PhC with μ_c1_ = 0.6 eV, μ_c2_ = 0.9 eV. The PhC nanocavity with resonant frequency around 175 THz was realized by introducing point defect into the 3 × 3 PhCs with an ultra-small size of 60 × 60 nm. Also, the bending wvaguide and the beam splitter are proposed by introducing the line defect in the crystal structure. The proposed graphene based PhC structure might find broad applications in the future high density plasmonic integrated circuit technique.
